# Autoimmune epilepsy due to *N*-methyl-d-aspartate receptor antibodies in a child: a case report

**DOI:** 10.1186/s13256-021-03117-5

**Published:** 2021-10-20

**Authors:** Jithangi Wanigasinghe, Thashi Chang

**Affiliations:** 1 Department of Paediatrics, Faculty of Medicine, University of Colombo, Colombo, Sri Lanka; 2 Department of Clinical Medicine, University of Colombo, Colombo, Sri Lanka

**Keywords:** Autoimmune epilepsy, NMDAR antibodies, Rituximab, Sri Lanka

## Abstract

**Introduction:**

Seizures of autoimmune etiology may occur independent of or predate syndromes of encephalitis. We report a child with “pure” autoimmune epilepsy followed up for 7 years to highlight long-term effects of this epilepsy and the importance of early initiation and appropriate escalation of immunosuppression to achieve a good long-term outcome.

**Case presentation:**

A previously healthy 5-year-old Sri Lankan boy presented with acute, frequent, brief focal seizures of temporal-lobe semiology without clinical and investigatory findings suggestive of central nervous system infection, tumor, structural abnormality, or metabolic causes. His epilepsy showed poor response to increasing doses and combinations of antiseizure medications. Further investigations detected *N*-methyl-d-aspartate receptor antibodies in serum, but not cerebrospinal fluid. Treatment with intravenous methyl prednisolone and maintenance on mycophenolate resulted in a rapid reduction, with seizure freedom achieved within 5–6 weeks. He relapsed when immunotherapy and anti seizure medications were reduced after seizure freedom for 24 months. This, and subsequent relapses, showed poor response to modification of anti-seizure medications, but treatment with immunotherapy (methyl prednisolone and rituximab) achieved complete seizure freedom. At 7-years of follow-up, he remains free of seizure for over 3 years, and has average academic performance and satisfactory quality of life.

**Conclusions:**

Autoimmune epilepsy is a recognized independent entity. Diagnostic criteria have been suggested for its early recognition and confirmation of diagnosis. Early diagnosis and initiation of immunosuppression, with prompt escalation of treatment when necessary, remains key to good patient outcome.

## Background

Antibodies targeting neuronal surface proteins (NSAbs) are increasingly recognized in autoimmune central nervous system (CNS) disorders in which seizures are the main or an important feature. *N*-methyl-d-aspartate receptor (NMDAR) encephalitis and NSAb-associated limbic encephalitis are the two leading syndromes within this group [1]. However, it has been increasingly recognized that seizures of autoimmune etiology may occur independent of or predate syndromes of encephalitis [2, 3]. It is considered specifically in those with drug-resistant epilepsies and epilepsies of unknown etiology [4]. The International League Against Epilepsy (ILAE) in their 2017 classification identified autoimmunity as one of the etiological subcategories of epilepsy [5]. The potential for effective treatment with immune therapy calls for its consideration in all patients presenting with poorly controlled epilepsy without a known etiology or a recognized epilepsy syndrome. We reported the first case of NMDAR-antibody encephalitis in Sri Lanka in 2012 [6] and now report the first case of autoimmune epilepsy (AEp) from Sri Lanka, a resource-restricted setting. We discuss the initial presentation and subsequent long-term epilepsy and neurocognitive outcome over the 7-year follow-up. We wish to highlight the importance of early recognition and treatment, and document the need for prolonged therapy in some patients.

### Case history

A 5-year-old, previously well, Sri Lankan boy presented in 2014 with recurrent seizures manifesting as staring episodes with behavioral arrest, progressively increasing from 10 to about 40 seizures a day. Some of the seizures were associated with autonomic features such as retching, vomiting, and tachycardia. In between seizures, his behavior fluctuated between normalcy, irritability, and increased sleepiness. Apart from two bouts of loose stools at the start of the illness, he remained afebrile without features of meningeal irritation such as neck stiffness or Kernig’s sign of papilledema. His cranial nerves, motor and sensory system, and cerebellar examination was normal. Glasgow Coma Scale score was 15/15. He did not have any abnormal movements or psychiatric manifestations. In between seizures, his pulse rate ranged from 90 to 100 beats per minute and blood pressure was 90/60 mmHg. His hematological and biochemical tests included full blood count (11.3 × 10^3^ μ/L) with normal differential), C-reactive proteins (< 5.0 mg/L), erythrocyte sedimentation rate (10 mm/hour), calcium (9.2 mg/dL), magnesium (1.9 mg/dL), and serum sodium (136 mmol/L) and potassium (4.3 mmol/L). Renal, liver, and thyroid functions [serum glutamic pyruvic transaminase (SGPT) 14 U/L, serum glutamic oxaloacetic transaminase (SGOT) 33 U/L, and blood urea 14 U/L], serum creatinine (36 μmol/L), triiodothyronine (T3) (3.44 ng/dL), and thyroxine (T4) (1.2 ng/dL) were normal. In his very first electroencephalogram, only intermittent slowing with theta and delta activity over right temporal and occipital region was noted. Subsequent video monitoring identified seizures with staring, grimace, and versive head movements. Ictal recording showed bilateral attenuation of background, evolving to fast activity and rhythmic theta over temporal, and then frontal region over the right side. No delta brush was noted in these records. Computerized tomography and subsequent magnetic resonance imaging (MRI) of the brain were normal. Although he was afebrile, a lumbar puncture was performed considering the possibility of a CNS infection. Cerebrospinal fluid (CSF) analysis did not show a pleocytosis, and protein level was within normalcy (22/mm^3^ lymphocytes, 1/mm^3^ polymorphs, sugar of 3.9 mmol/L, and protein of 30 mg/dL); bacterial antigens were negative, and culture yielded no growth. Herpes simplex viral screen was negative. Stool analysis and culture were negative for infection. The initial electroencephalogram revealed intermittent slow background activity, particularly in the temporal and occipital regions. No focal or generalized epileptic discharges were recorded. No extreme delta brush was noted. He was the firstborn, with birth weight of 3.45 kg at 38 weeks. He did not have any major illness prior to this admission. There was no significant family history of note. He was only attending mainstream school in grade 2 at the time of onset of his illness.

He was initially treated with cefotaxime (50 mg/kg/dose 6-hourly) and aciclovir (250 mg/m^2^ 8-hourly), which were discontinued when the CSF findings were normal, including herpes simplex virus 1 (HSV-1= viral polymerase chain reaction (PCR) in CSF. He was commenced simultaneously on intravenous followed by multiple oral antiseizure medications (ASMs). These were phenobarbitone given intravenously (20 mg/kg) and levetiracetam, carbamazepine, and clobazam given in escalating doses gradually via oral route. The dosages were 30 mg/kg/day, 18 mg/kg/day, and 5 mg/day, respectively, the seizures progressively reduced in frequency and duration to 10–15 brief seizures per day. He was discharged from hospital after 2 weeks on three ASMs, albeit with infrequent seizure recurrences.

Although ASMs were increased in dose and in different combinations, there was no improvement in seizure control. Within the next 4 weeks, escalation up to 30–40 brief seizures per day, both with and without altered consciousness was noted. His serology at this point was negative for antinuclear, thyroglobulin, and thyroid peroxidase antibodies. Serum lactate level was 1.2 mmol/L. Facilities for advanced metabolic screening or genetic panels for epilepsy were unavailable. A repeat CSF analysis showed normal lactate and glucose values. However, examination of paired serum and CSF revealed presence of *N*-methyl-d-aspartate receptor (NMDAR) antibodies in serum but not in CSF (live cell-based assay, Oxford, UK). At this point, on retrospective analysis, we note that he fulfilled Antibody Prevalence in Epilepsy and Encephalopathy (APE2) score of 4 in the proposed autoimmune epilepsy diagnostic criteria described in 2017, in which a score of ≥ 4 predicts has a sensitivity of 98% and a specificity of 85% for prediction of neural specific antibody seropositivity [7].

He was treated with intravenous methylprednisolone 30 mg/kg/day for 3 days, which resulted in significant reduction of seizure frequency after the first pulse, followed by complete freedom of seizures after the second pulse a month later. He was maintained on oral prednisolone (2 mg/kg/day), followed by transition to mycophenolate mofetil (MMF) at a starting dosage of 600 mg/m^2^ twice daily. Serial ultrasonography excluded a testicular teratoma. On retrospective analysis, he can be considered to have demonstrated a Response to Immunotherapy in Epilepsy and Encephalopathy (RITE2) score of 8, which confirms his condition to be a definite autoimmune epilepsy.

**Fig. 1:**
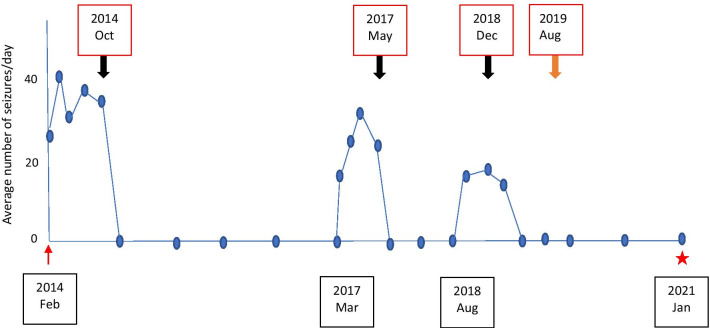
The temporal profile of seizure frequency since presentation and its response to immunotherapy. The *x*-axis indicates the time course from presentation (red arrow) to last review (red star) with time points of relapses. Time points of immunotherapy (black arrows, intravenous methylprednisolone; orange arrow, rituximab) are indicated above the graph

He remained seizure free for 3 years and continued schooling with no concerns, and his behavior continued to be normal. Subsequent two electroencephalograms done annually showed no abnormality. However, he relapsed when attempting to tail off ASMs and MMF (March 2017). The repeat MRI of brain reviewed carefully remained normal without evidence of focal cortical dysplasia, and cortical or subcortical hyperintensities. The temporal lobes remained unchanged in hyperintensity or in size. Intensified treatment with levetiracetam (gradually up to 50 mg/kg/day), carbamazepine (20 mg/kg/day), and clobazam (10 mg/day) was ineffective, but pulsed intravenous steroids (methyl prednisolone 30 mg/kg/day for 3 days) given two times with 1-month interval resulted in complete resolution of seizures. One year later, while on regular MMF and ASMs, he relapsed with similar seizures (August 2018). Electroencephalography (EEG) at this point showed interictal epileptic activity over the right pericentral (C4) region and brief subclinical ictal activity consisting of rhythmic theta over the F4, Fz, and C4 region. Repeat serum and CSF examination detected persistence of NMDAR antibodies in low titers in the serum. Seizure resolution was achieved once again with pulsed high-dose intravenous steroids. To achieve longer remission, he was treated with rituximab (August 2019) given as four weekly doses of 375 mg/m^2^. Currently, he is 12 years old and has remained seizure-free for more than 2 years since last relapse in 2018 (Fig. 1). He schools in an age-appropriate grade with average academic performance comparable to his peers in school. His quality of life, assessed using the Sri Lankan Health Related Quality of Life Index for school children (SLHRQ-S), an age-specific, primary caregiver proxy rated, validated questionnaire for Sri Lankan children with epilepsy [8], demonstrated a mean score of 84.13. Individual scores were 83.3 (physical), 80.8 (psychological), and 88.3 (social) for the respective domains. These were within normal range of the validation scores.

## Discussion

This report narrates the progression of a child with poorly controlled epilepsy, subsequently confirmed a definitive diagnosis of autoimmune epilepsy without encephalopathy, treated with progressive courses of immunotherapy resulting in normal childhood and intellectual growth with satisfactory quality of life. Early institution of immunotherapy and its timely escalation to achieve long-term remission is emphasized.

In the 2017 ILAE concept paper, “Epilepsy of immune aetiology” was considered for persons in whom “epilepsy was directly from an immune disorder in which the seizures are a core symptom of the disorder” [5]. Criteria and supportive features to diagnose AEp are presented in Table 1 [9]. Those with supportive criteria for AEp are divided into four groups: “definite,” “possible,” “probable,” and “unlikely” [10], which was subsequently modified to include a fifth category as “unknown AEp” [2]. In our patient, the diagnosis fulfilled “definite” criteria. Subsequent description of a predictive model in 2017 aided diagnosis, treatment, and prognostication of autoimmune epilepsy [11]. In this, an RITE2 score of ≥ 7 is associated with definite diagnosis of autoimmune epilepsy, with sensitivity of 88% and specificity of 84%; our patient had scored 8.

**Table 1:** Diagnostic criteria for autoimmune epilepsy (AEp) [2]

Diagnosis of AEp requires the presence of the following two clinical criteria:
1. Acute or subacute (< 12 weeks) onset of symptoms, and
2. Exclusion of other causes (CNS infection, trauma, toxic, tumor, metabolic, previous CNS disease)
The presence of at least one of the following supportive features would strengthen the suspicion of AEp:
1. The presence of a well-defined clinical syndrome such as NMDAR or limbic encephalitis;
2. CNS inflammation manifested by at least one of the following:
a. CSF pleocytosis (defined as > 5 white cells/mm^3^) or presence of oligoclonal bands, elevated IgG index, or elevated neopterin (defined as > 30 nM);
b. MRI abnormality compatible with an inflammatory or autoimmune encephalitis including increased signal in the mesio-temporal lobe (LE-like syndrome); or
c. Inflammatory neuropathology on biopsy
3. History of other antibody-mediated conditions (for example myasthenia gravis), organ-specific autoimmunity, or other autoimmune disorders; or
4. Response to immunotherapy

*AEp* Autoimmune epilepsy,* CNS* Central Nervous System,* NMDAR* N-methyl-D-aspartate receptor,* CSF* Cerebrospinal fluid,* IgG* Immunoglobulin,* MRI* Magnetic Resonance Imaging,* LE* Limbic encephalitis

The role of immunity in epileptogenesis is related to different types of immunity [12]. A specific role has been shown for innate immunity, where activation of glial cells occurs owing to release of inflammatory molecules in brain injury, convulsive events, and some genetic epilepsies [13]. Similarly, the peripheral immune system mediated by lymphocytes has been shown to play a role in disruption of the blood–brain barrier [14]. Additionally, an increasing number of clinical and neuropathological observations have shown that activation of inflammatory processes occurs in a variety of focal epilepsies without infectious or immune-mediated etiology [12].

In our patient, it is the adoptive immunity that plays a role in epileptogenicity. There has been a massive expansion of knowledge, particularly over the past two decades, with demonstration of causality between autoimmunity and several seizure-related diseases [13, 15]. Antibodies described in AEp include antibodies directed against intracellular proteins, such as glutamic acid decarboxylase 65-kilodalton isoform (GAD65), and NSAb directed against voltage-gated potassium channel (VGKC)-complex proteins, glutamate [NMDA and α-amino-3-hydroxy-5-methyl-4-isoxazolepropionic acid (AMPA)], and gamma-amino butyric acid-A (GABAA) and gamma-amino butyric acid-B (GABAB) receptors [13, 16]. Antibody-negative AEp has been reported in about 36% of patients [17]. AEp was initially described as part of a syndrome of encephalitis, but has now been recognized as an entity of its own, as seen in our patient who did not develop encephalitis over a period of 7 years of follow-up. It could be argued that early initiation of immunotherapy may have prevented the progression to encephalitis, but this appears less likely given the long follow-up and quiescent disease during periods of waning immunotherapy.

Immunotherapy-responsive, CSF-negative but serum-positive NMDAR-antibody encephalitides have been previously reported, with the observation that serum antibodies were always higher than CSF in paired samples, and that, with time, previously present CSF antibodies may diminish with preserved serum antibodies [18, 19]. Indeed, this may have been the case in our patient, in whom NMDAR antibodies were tested late during the first presentation, while in the second presentation the serum antibody response being of low titer may have had a paired CSF titer that was not detectable. Furthermore, it has been hypothesized that immunoprecipitation of the antibody at the blood–brain barrier may account for this discrepancy of seropositive but CSF-negative autoimmune encephalitis in some patients [20].

Features that would suggest an autoimmune etiology in epilepsy include young age at onset, previous normal health, multiple focal seizures occurring several times a day, temporal lobe semiology, and some specific seizure semiologies such as faciobrachial dystonia and paroxysmal dizzy spells, poor response to ASM, and negative brain imaging [17, 21]. Seizure semiologies suggesting AEp include usually brief, focal seizures, with or without retained awareness, rapid recovery with minimal post-ictal features, and occur with higher frequency in sleep. They may be multifocal and may have changing semiologies [21]. Other factors to suspect AEp include personal or family history of autoimmune disorders, or recent or past neoplasia [21]. Literature on specifics of treatment of AEp and its long-term outcome are rare but immunotherapy, if instituted early, has shown to result in good outcomes in epilepsies associated with NSAbs [21]. Type of therapy is mostly guided by recommendations available for autoimmune encephalitis, that is, steroids, plasmapheresis, and intravenous immunoglobulins as first-line immunotherapy; rituximab, cyclophosphamide, MMF, and azathioprine as second line; and tocilizumab and bortezomib as third-line therapy [22]. Treatment strategies for new-onset refractory status epilepticus (NORSE) have been described. Prospective studies are needed for establishing treatment algorithms specific for autoimmune epilepsy. Initiation of immune therapy early for AEp (within 6 months of disease onset) is shown to result in favorable seizure control [11]. This was the case in our patient. Larger reports of long-term outcomes will be useful to understand the disease evolution and its behavior with immune therapy.

## Conclusion

Our case report highlights the importance of early diagnosis of AEp, early treatment with immunotherapy, long-term clinical follow-up, and timely escalation and continuation of immunotherapy in achieving a good patient outcome, retaining intellectual development, and quality of life.

## Data Availability

Not applicable.

## References

[1] Graus F, Titulaer MJ, Balu R, Benseler S, Bien CG, Cellucci T, Cortese I, Dale RC, Gelfand JM, Geschwind M,* et al*. A clinical approach to diagnosis of autoimmune encephalitis. Lancet Neurol. 2016;15(4):391–404. 10.1016/S1474-4422(15)00401-9 PMC5066574 26906964

[2] Suleiman J, Brilot F, Lang B, Vincent A, Dale RC. Autoimmune epilepsy in children: case series and proposed guidelines for identification. Epilepsia. 2013;54(6):1036–45. 10.1111/epi.12142 23551014

[3] Thompson J, Bi M, Murchison AG, Makuch M, Bien CG, Chu K, Farooque P, Gelfand JM, Geschwind MD, Hirsch LJ,* et al*. The importance of early immunotherapy in patients with faciobrachial dystonic seizures. Brain. 2018;141(2):348–56. 10.1093/brain/awx323 PMC5837230 29272336

[4] Iorio R, Assenza G, Tombini M, Colicchio G, Della Marca G, Benvenga A, Damato V, Rossini PM, Vollono C, Plantone D,* et al*. The detection of neural autoantibodies in patients with antiepileptic-drug-resistant epilepsy predicts response to immunotherapy. Eur J Neurol. 2015;22(1):70–8. 10.1111/ene.12529 25112548

[5] Scheffer IE, Berkovic S, Capovilla G, Connolly MB, French J, Guilhoto L, Hirsch E, Jain S, Mathern GW, Moshe SL,* et al*. ILAE classification of the epilepsies: position paper of the ILAE Commission for Classification and Terminology. Epilepsia. 2017;58(4):512–21. 10.1111/epi.13709 PMC5386840 28276062

[6] Wanigasinghe J, Chang T, Vincent A. Treatment-responsive, reversible, autoimmune encephalitis in a child. Ceylon Med J. 2012;57(2):90–1. 10.4038/cmj.v57i2.4466 22772791

[7] Dubey D, Pittock SJ, McKeon A. Antibody prevalence in epilepsy and encephalopathy score: increased specificity and applicability. Epilepsia. 2019;60(2):367–9. 10.1111/epi.14649 30727035

[8] Murugupillai R, Wanigasinghe J, Muniyandi R, Arambepola C. Development of a pilot health related quality of life tool for Sri Lankan children with epilepsy. Sri Lanka J Child Health. 2016;45(3):163.

[9] Suleiman J, Brenner T, Gill D, Brilot F, Antony J, Vincent A, Lang B, Dale RC. VGKC antibodies in pediatric encephalitis presenting with status epilepticus. Neurology. 2011;76(14):1252–5. 10.1212/WNL.0b013e3182143552 21464429

[10] Zuliani L, Graus F, Giometto B, Bien C, Vincent A. Central nervous system neuronal surface antibody associated syndromes: review and guidelines for recognition. J Neurol Neurosurg Psychiatry. 2012;83(6):638–45. 10.1136/jnnp-2011-301237 PMC3348613 22448032

[11] Dubey D, Singh J, Britton JW, Pittock SJ, Flanagan EP, Lennon VA, Tillema JM, Wirrell E, Shin C, So E,* et al*. Predictive models in the diagnosis and treatment of autoimmune epilepsy. Epilepsia. 2017;58(7):1181–9. 10.1111/epi.13797 28555833

[12] Vezzani A, Lang B, Aronica E. Immunity and inflammation in epilepsy. Cold Spring Harb Perspect Med. 2015;6(2):a022699. 10.1101/cshperspect.a022699 PMC4743070 26684336

[13] Geis C, Planaguma J, Carreno M, Graus F, Dalmau J. Autoimmune seizures and epilepsy. J Clin Invest. 2019;129(3):926–40. 10.1172/JCI125178 PMC6391086 30714986

[14] Wright S, Vincent A. Pediatric autoimmune epileptic encephalopathies. J Child Neurol. 2017;32(4):418–28. 10.1177/0883073816685505 28056633

[15] McKnight K, Jiang Y, Hart Y, Cavey A, Wroe S, Blank M, Shoenfeld Y, Vincent A, Palace J, Lang B. Serum antibodies in epilepsy and seizure-associated disorders. Neurology. 2005;65(11):1730–6. 10.1212/01.wnl.0000187129.66353.13 16344514

[16] Irani SR, Bien CG, Lang B. Autoimmune epilepsies. Curr Opin Neurol. 2011;24(2):146–53. 10.1097/WCO.0b013e3283446f05 21358545

[17] Lv RJ, Ren HT, Guan HZ, Cui T, Shao XQ. Seizure semiology: an important clinical clue to the diagnosis of autoimmune epilepsy. Ann Clin Transl Neurol. 2018;5(2):208–15. 10.1002/acn3.520 PMC5817820 29468181

[18] Zandi MS, Paterson RW, Ellul MA, Jacobson L, Al-Diwani A, Jones JL, Cox AL, Lennox B, Stamelou M, Bhatia KP,* et al*. Clinical relevance of serum antibodies to extracellular N-methyl-d-aspartate receptor epitopes. J Neurol Neurosurg Psychiatry. 2015;86(7):708–13. 10.1136/jnnp-2014-308736 PMC6055984 25246644

[19] Warren N, Swayne A, Siskind D, O’Gorman C, Prain K, Gillis D, Blum S. Serum and CSF Anti-NMDAR antibody testing in psychiatry. J Neuropsychiatry Clin Neurosci. 2020;32(2):154–60. 10.1176/appi.neuropsych.19030079 31530118

[20] Castillo-Gomez E, Kastner A, Steiner J, Schneider A, Hettling B, Poggi G, Ostehr K, Uhr M, Asif AR, Matzke M,* et al*. The brain as immunoprecipitator of serum autoantibodies against N-methyl-d-aspartate receptor subunit NR1. Ann Neurol. 2016;79(1):144–51. 10.1002/ana.24545 26505629

[21] Quek AM, Britton JW, McKeon A, So E, Lennon VA, Shin C, Klein C, Watson RE Jr, Kotsenas AL, Lagerlund TD,* et al*. Autoimmune epilepsy: clinical characteristics and response to immunotherapy. Arch Neurol. 2012;69(5):582–93. 10.1001/archneurol.2011.2985 PMC3601373 22451162

[22] Stingl C, Cardinale K, Van Mater H. An update on the treatment of pediatric autoimmune encephalitis. Curr Treatm Opt Rheumatol. 2018;4(1):14–28. 10.1007/s40674-018-0089-z PMC5957495 29780690

